# Psychometric properties of the Problematic Use of Social Networks (PUS) scale in Arabic among adolescents

**DOI:** 10.1371/journal.pone.0291616

**Published:** 2023-09-14

**Authors:** Sahar Obeid, Covadonga González-Nuevo, Álvaro Postigo, Abir Sarray El Dine, Vanessa Azzi, Diana Malaeb, Souheil Hallit

**Affiliations:** 1 School of Arts and Sciences, Social and Education Sciences Department, Lebanese American University, Jbeil, Lebanon; 2 Department of Psychology, University of Oviedo, Oviedo, Spain; 3 Department of Biomedical Sciences, School of arts and Sciences, Lebanese International University, Beirut, Lebanon; 4 School of Medicine and Medical Sciences, Holy Spirit University of Kaslik, Jounieh, Lebanon; 5 School of Pharmacy, Gulf Med Uni, Ajman, United Arab Emirates; 6 Psychology Department, College of Humanities, Effat University, Jeddah, Saudi Arabia; 7 Applied Science Research Center, Applied Science Private University, Amman, Jordan; 8 Research Department, Psychiatric Hospital of the Cross, Jal Eddib, Lebanon; Canadian University Dubai, UNITED ARAB EMIRATES

## Abstract

**Background:**

The Problematic Use of Social Networks (PUSN) scale assesses for the first time the comparative use of social networks along with addictive tendencies. However, it has only been validated in a Spanish sample. This study aimed to evaluate the psychometric properties of the Arabic version of the PUSN (PUSN-Ar) among Lebanese adolescents.

**Methods:**

A total of 379 adolescents aged between 15 to 18 years (*M* = 16.07 years; 64.9% females) participated in this cross-sectional study conducted between January and May 2022. The problematic use of social networks—SNS (PUSN) scale yields of two subscales: addiction-related consequences of SNS use (SNS-ARC) and negative social comparison (SNS-NSC). The Beirut Distress Scale, the Smartphone Addiction Scale and the Jong-Gierveld Loneliness Scale were used to assess psychological distress, smartphone addiction and loneliness respectively.

**Results:**

The PUS-Ar scale exhibited a two-dimensional structure (Comparative Fit Index [CFI] = .97; Root Mean Square Error of Approximation [RMSEA] = .08), consistent with the original scale. Both subscales, Negative Social Comparison (α = .96) and Addictive Consequences (α = .92), demonstrated excellent reliability. Additionally, measurement invariance was verified between males and females at the configural, metric and scalar levels. No significant difference was found between males and females in terms of SNS-NSC (20.75 ± 10.15 vs 21.09 ± 9.51; *t*(377) = -.32; *p* = .751) and SNS-ARC (19.08 ± 8.61 vs 19.76 ± 6.77; *t*(377) = -.79; *p* = .430). The SNS-ARC subscale was more correlated to smartphone addiction than the SNS-NSC (*r* = .73; *p* < .001 vs *r* = .54; *p* < .001) whereas both subscales were significantly associated with more stress and more loneliness.

**Conclusion:**

The data collected in this study provided support for all the hypotheses formulated. Consequently, the PUS-Ar was deemed a suitable scale to measure problematic SNS among Lebanese adolescents. The PUS-Ar is currently available to researchers for use in evaluating PSNSU in Lebanon. However, it is important to note that further research is needed to explore its applicability and generalizability across different populations and contexts.

## Background

Our lives have become increasingly dependent on Social Networking Sites (SNS), playing a vital role in communication and information sharing [1]. These platforms enable individuals to connect and socialize with family, friends, and colleagues, fostering a feeling of connection and belonging [2]. The global adoption of SNS is increasing, with more than 2 billion users worldwide [3]. In terms of SNS usage, Western countries lead with social media access rates of 70% and 66%, respectively [3]. YouTube, Instagram and Snapchat are the most popular SNS for American teens, 45% of teenagers claim to be online nearly constantly [4]. However, problematic use of SNS (PUSNS), defined as “using SNS for more than two hours per day”, tends to be potentially correlated with poor mental health, health-related, interpersonal, educational problems [5, 6], as well as smartphone addiction [7], and positively correlated with psychiatric disorder symptoms such as psychological distress, suicidal ideation [8], eating disorders [9], anxiety [10–12], loneliness [13], attention deficit hyperactivity disorder [14, 15], obsessive compulsive disorder and depression [16] among a minority of individuals [1, 17]. On the other hand, research revealed that PSNSU inclines to be negatively associated with life satisfaction [18, 19] and quality of life [20].

Problematic use of SNS (PSNSU), or SNS addiction [5], has been theoretically defined as “a disorder that does not encompass the ingestion of a psychoactive substance and shares criteria with behavioral addiction” [21]. In this line, previous authors [5] define SNS addiction *as “being overly concerned about SNSs*, *driven by a strong motivation to log on to or use SNSs*, *and to devote so much time and effort to SNSs that it impairs other social activities*, *education and/or occupation*, *interpersonal relationships*, *and/or psychological health and well-being”*. Regarding the prevalence of PSNSU, the rates fluctuate from 1.6% [22] to 12% in China [23].

Apart from gambling and gaming disorder [24, 25], PSNSU is not yet included in the Diagnostic and Statistical Manual of Mental Disorders, fifth edition, and is not officially recognized as a clinical disorder. However, researchers have argued that it may be considered as "other specified disorders due to addictive behaviors" in the eleventh edition of the International Classification of Diseases (ICD-11) [26].

In 2021, González-Nuevo et al. [27] developed and validated in Spain the problematic use of social networks—SNS (PUS) scale, the only self-report scale to assess the PSNSU so far. The scale consists of two subscales: addiction-related consequences of SNS use (SNS-ARC) and negative social comparison (SNS-NSC). The addictive use component captures the impact of SNS use on various aspects of daily life, while the negative social comparison component focuses on comparisons made on social networks that can lead to feelings of inferiority. Thus, this innovative tool enables a comprehensive evaluation of inappropriate SNS usage, encompassing both the consequences of such usage and the inclination to engage in relational comparisons through SNS [27]. The PUS scale, consisting of 18 items, demonstrated excellent psychometric properties and displayed higher reliability compared to other questionnaires assessing the addictive use of social networks (i.e. Social Media Disorder Scale (SMDS) [28], the Facebook Intrusion Questionnaire (FIQ) [29], the Bergen Facebook Addiction Scale (BFAS) [30] and the Bergen Social Media Addiction Scale (BSMAS) [16]).

Language and culture can influence the translation of a scale into another language, which can lead to unequal measurements, especially in the science/health field [31]. Therefore, our goal was to assess the psychometric properties of the Arabic version of the PUS (PUS-Ar) related to its factor structure, reliability and validity in a sample of Lebanese adolescents.

## Materials and methods

### Ethics approval and consent to participate

The study was conducted in accordance with the Declaration of Helsinki and was approved by the ethics committee of the School of Pharmacy at the Lebanese International University (approval number: 2021RC-048-LIUSOP). Informed consent was obtained from the parent and/or legal guardian for participants under age 16 before filling the survey. Submitting the form online was considered equivalent to obtaining a written informed consent.

### Study design and participants

This study employed a cross-sectional design and was performed between January to May 2022, involving 379 adolescents residing in Lebanon (aged 15 to 18 years) from various Lebanese governorates (*M* = 16.07 years; *SD* = 1.19; 64.9% females). The snowball technique was utilized to select our sample via a Google forms link. Before their enrollment, participants were informed of the study’s primary goals, and instructions for completing the questionnaire through online communication. Subsequently, we encouraged participants to recruit others in their social network, with an emphasis on achieving diversity in terms of their region of living within the Lebanese governorates and the specified age range for participation. No incentives or credits were offered for taking part in the study.

### Instruments

The questionnaire collected data about age and sex of responders, as well as the following scales:

#### Problematic Use of Social Networking Sites (PUSN) Scale

It is a self-report tool created to investigate the possible effects of Social Networking Sites (SNS) usage in terms of addiction-related outcomes and comparative usage [27]. The 18 items of the scale were graded using five categories (1 = completely disagree, 5 = completely agree). There are two subscales made up of the items: 8 items for SNS-ARC and 10 items for SNS-NSC. Higher scores indicate higher PSNSU in both domains (α = .95 for Negative Social Comparison and α = .90 for Addictive Consequences). The forward and backward translation method was applied to the PUSN scale following international guidelines [32]. The English version was translated to Arabic by a Lebanese translator who was completely unrelated to the study. Afterwards, a Lebanese psychologist with a full working proficiency in English, translated the Arabic version back to English. The initial and translated English versions were compared to detect and later eliminate any inconsistencies by a committee composed of the research team and the two translators [33, 34]. A pilot study was conducted on 20 persons before the start of the official data collection to make sure all questions are well understood; no changes were done consequently [35].

The Beirut Distress Scale (BDS-10) validated in Lebanon [36] (α = .90), the Smartphone Addiction Scale (SAS) [37, 38], validated in Lebanon [39] (α = .90), and the validated Arabic version of the Jong-Gierveld Loneliness Scale [40] (α = .76) were used to assess psychological distress, smartphone addiction and loneliness respectively.

### Data analysis

To answer our objectives, we conducted a confirmatory factor analysis (CFA). The method of estimation used was Unweighted Least Squares estimates with standard errors and a mean- and variance- adjusted chi-square test (ULSMV). The indices of fit were Comparative Fit Index (CFI) and Root Mean Square Error of Approximation (RMSEA). A good fit was observed if CFI > .95 and RMSEA < .08 [41]. Corrected item-test correlations were calculated to assess the discrimination indices of the items, with values above 0.2 considered acceptable [42, 43]. Reliability was evaluated using Cronbach’s α [44] and McDonald ‘s ω [45].

We used the Multigroup Confirmatory Factor Analysis (MG-CFA) process to examine measurement invariance between sex, which occurs at different levels; configural, metric, and scalar invariance. Measurement invariance was studied between Lebanese men and women. For estimating parameters, we used Weighted Least Squares, and the Mean and Variance (WLSMV) adjusted estimation method, as recommended for categorical data [46, 47]. As a base model, we performed various CFA’s under a bidimensional structure to examine the fit of the instrument for each group separately. To accept measurement invariance, the reduction in CFI must be below .01 (ΔCFI<-.01) [48].

To examine the validity of the PUS instrument concerning other variables, we calculated Pearson correlations between the new instrument and all other scales previously described: a) The Smartphone Addiction Scale (SAS) b) Jong-Gierveld Loneliness Scale, and c) Beirut Distress Scale (BDS-10).

We calculated the Average Variance Extracted (AVE) to verify the convergent validity, using the method described in Fornell and Larcker [49]; values ≥ .50 are satisfactory. Discriminant validity between the two PUSN subscales was deemed confirmed if the AVE values for each scale were higher than the square of the correlation between them [49].

The SPSS v.24 statistics package (IBM Corp, 2016) was used for the remaining analyses. Skewness and kurtosis values ranging between -1 and +1 indicated that the scores for the two PUS subscales were normally distributed [50]. Pearson correlations, Student t test and ANOVA were performed. Reliability coefficients were calculated using the FACTOR 10.10.02 software [51]. Finally, the CFA and measurement invariance between sexes and countries were conducted using the Mplus8 program [46].

## Results

### Evidence based on internal structure and reliability

A CFA was conducted to test the original two-factor model. Factor loadings and discrimination indices were very high in both factors (Table 1). Both subscales showed excellent internal consistency (SNS-NSC: α = .96 / ω = .96; SNS-ARC: α = .92 / ω = .92). The CFI and RMSEA values demonstrated a good fit (CFI = .97; RMSEA [90% CI] = .08 [.07 - .09]).

**Table 1:** Factor loadings and discrimination index for the items in the PUS-Ar questionnaire.

	Items	F. L. Negative Social Comparison	F. L. Addictive Consequences	*DI*	*M*	*SD*
1	When I see content from influencers or celebrities, I feel inferior	.86		.78^1^	2.04	1.15
2	When I see content from my friends or people I know, I feel inferior	.80		.73^1^	2.13	1.13
3	When I’m not on social networks, I feel an impulse to go online that is hard to resist		.78	.68^2^	2.33	1.17
4	When I publish my content, I worry that it will be made fun of	.79		.71^1^	2.23	1.24
5	When I see what celebrities or influencers publish, I feel bad about myself	.86		.78^1^	1.83	1.10
6	When I see what my contacts publish, I feel that they have a better life than me	.87		.81^1^	2.18	1.25
7	I spend too much time using social networks		.66	.65^2^	2.88	1.24
8	I don’t get enough sleep because of using social networks		.76	.68^2^	2.41	1.22
9	I have tried to spend less time on social networks, but I have not been successful		.80	.71^2^	2.49	1.24
10	Most of my friends and people I know on social networks are happier than I am	.82		.74^1^	2.07	1.12
11	I compare myself with other people who I think are better than me on social networks	.89		.82^1^	2.01	1.21
12	I worry that my posts will not have enough positive interactions	.88		.79^1^	2.19	1.21
13	I worry a lot about what people might think of my content	.85		.77^1^	2.13	1.20
14	I feel lonely when I see what my contacts post on social networks	.90		.83^1^	2.14	1.21
15	My academic or work performance has declined because of using social networks		.80	.68^2^	2.23	1.23
16	People close to me have complained because I use social networks too much		.79	.66^2^	2.19	1.20
17	I feel that if I am not connected to social networks, I am missing out on something		.75	.65^2^	2.42	1.23
18	Using social networks, I lose track of time and ignore important tasks I have outstanding		.81	.71^2^	2.59	1.27

*Note*. n = 379; F.L. = Factor Loadings

^1^ = Discrimination Index of the items in the first factor, called Negative Social Comparison

^2^ = Discrimination Index of the items in the second factor, called Addictive Consequences

### Measurement invariance across sex

We were able to show measurement invariance for PUS-Ar scores at configural, metric, and scalar levels between males and females (Table 2). No significant difference was found between males and females in terms of SNS-NSC (20.75 ± 10.15 vs 21.09 ± 9.51; *t*(377) = -.32; *p* = .751) and SNS-ARC (19.08 ± 8.61 vs 19.76 ± 6.77; *t*(377) = -.79; *p* = .430).

**Table 2:** Measurement invariance for PUS-Ar based on sex in Lebanon.

		χ^2^ (df)	CFI	RMSEA [90% CI]	ΔCFI
Women (*n* = 246)		437.817 (134)	.958	.096 [.086 - .106]	-
Men (*n* = 133)		343.111 (134)	.977	.108 [.094 - .123]	-
	Configural Invariance	788.943 (268)	.968	-	-
	Metric Invariance	807.941 (284)	.968	-	0
	Scalar Invariance	781.306 (336)	.972	-	.004

*Note*. χ^2^ = Satorra-Bentler chi-square; df = degrees of freedom; RMSEA = root mean square of approximation; CFI = comparative fit index; ΔCFI = CFI change

### Concurrent validity

The two subscales of the PUS-Ar were highly correlated to each other (*r* = .72). The addictive consequences subscale was more correlated to smartphone addiction, whereas both subscales were significantly associated with more stress and more loneliness (Table 3).

**Table 3:** Correlation between problematic SNS use, smartphone addiction, stress and loneliness.

	Negative social comparison	Addictive consequences
Negative social comparison	1	
Addictive consequences	.72***	1
Smartphone addiction	.54***	.73***
Stress	.48***	.45***
Loneliness	.52***	.42***

Numbers in the table refer to Pearson correlation coefficients.

***p < 0.001

Convergent validity was acceptable since the SNS-NSC (AVE = .59) and SNS-ARC (AVE = .72) factors had AVE values above .5. Discriminant validity was also verified (r^2^xy = 0.52).

## Discussion

The fit indices from the CFA results allow us to replicate the original structure of the PUS scale from the original Spanish study [27], which indicates a strong factorial validity. Furthermore, the results showed no difference between males and females, similar to the Spanish results [27].

Moreover, the Cronbach’s alpha coefficients of the PUS-Ar subscales were excellent (α = .96 and .92 respectively), reflecting a high internal consistency. Those numbers are very comparable to the ones obtained in the Spanish study (α = .96 for SNS-NSC and α = .92 for SNS-ARC) [27]. Again, the reliability was greater than other tools that evaluate the addictive aspect of SNS (e.g. short version of the SMDS [28], FIQ [29], BFAS [30] and BMSAS [16].

Regarding concurrent validity, both PUS-Ar subscales scores correlated positively with psychological distress, in agreement with previous findings [11, 52–54]. The SNS-NSC was more associated with stress than SNS-ARC, reflecting the importance of assessing the social comparison aspect of social networking. Xu and Tan [55] reasoned that PSNSU befalls when a subject considers SNS use as a strategy to reduce the level of stress. The score was also positively correlated with loneliness, corroborating previous findings from Lebanon [13].

Concerning the convergent validity, the findings revealed a more positive association between the Addictive Consequences subscale and smartphone addiction over its association with the Negative Social Comparison subscale, in agreement with a previous study stating that SNS is a strong predictor of smartphone addiction [7]. A previous study [56] revealed that the smartphone devices addiction is greater than addiction to SNS.

With these findings, we conclude that the PUS-Ar is a valid and reliable instrument to measure, to our knowledge, for the first time, the comparative and addictive use of social networks in a Lebanese sample. In addition, this validation has been carried out in a sample of adolescents, which is an important novelty, since the younger the age, the higher the PSNSU. PUS-Ar also demonstrated measurement invariance across sex, so that comparisons between sexes can be made in a reliable way. As a result, it will be possible to advance in the study of the PSNSU with an appropriate instrument for its measurement and will allow international studies in which different cultures can be compared [1].

### Limitations

Due to the cross-sectional design of this study, the ability to establish causality is limited. Therefore, we cannot determine the causality between PSNSU, stress, loneliness, and smartphone addiction. As a cross-sectional study, there is a possibility of recall and information biases, due to the overestimation of responses or misunderstanding of certain questions. Symptoms were self-reported and not evaluated by healthcare professionals, making them subjective in nature. Moreover, selection bias may have occurred due to the refusal rate and the overrepresentation of females in the sample. Therefore, our results cannot be generalized as the sample consisted of adolescents recruited through the snowball technique. Cultural adaptation of the PSNSU scale was not done in this study.

## Conclusion

In this study, the main objective was to establish the validity of the PUS-Ar by investigating its factor structure, concurrent validity, and internal consistency. The data collected in this study provided support for all the hypotheses formulated. Consequently, the PUS-Ar was deemed a suitable scale to measure problematic SNS among Lebanese adolescents. The PUS-Ar is currently available to researchers for use in evaluating PSNSU in Lebanon. Regarding the practical implications, this instrument will allow clinicians assess the level of PSNSU and undertake measures to minimize or prevent it.
